# Introducing 'global health methodology research' a knowledge sharing platform open to all

**DOI:** 10.1186/1745-6215-14-S1-P35

**Published:** 2013-11-29

**Authors:** Francois van Loggerenberg, Mike Clarke, Tamzin Furtado, Liam Boggs, Nicola McHugh, Samuel Franzen, Trudie Lang

**Affiliations:** 1 The Global Health Network, Oxford University Centre for Tropical Medicine, University of Oxford, Oxford, UK; 2 Centre for Public Health, Queen's University, Belfast, Northern Ireland, UK

## 

Clinical trials need evidence-led improved methods and operations and this data should be derived globally. Global Health Methodology Research has been set up as a free and open facility for a growing community of researchers who are interested in supporting the generation of more and better evidence to drive improvements in health research methods in varied settings. Research regulations are increasingly applied globally, given that many studies are conducted as multi-centre trials that operate across highly varied geographical regions. Across all settings, there are increasing calls for changes in how trials are conducted. This is in response to international recognition that clinical research has become too expensive and cumbersome and that would-be investigators are put off from engaging in research because they perceive that operating studies is too difficult and expensive, with needlessly complex and restrictive regulations.

Here we are seeking to build an open collaboration of groups and individuals who want to engage in developing research methodology studies. Whilst there are differences in the environment and resources between high income and developing countries, the challenges faced when operating trials are largely similar and much can be learnt from operating trials in challenging settings. This platform has been established from http://www.globalhealthtrials.org as a dedicated space to discuss, develop and indeed conduct clinical research methodology studies. It is clear that there is a need to conduct methodology research around and nested within studies to guide improvements to design and operation of studies.

